# Insertional Achilles Tendinopathy Treated With Botulinum Toxin: A Case Report

**DOI:** 10.7759/cureus.66641

**Published:** 2024-08-11

**Authors:** Alexander Kim, Alexander Knobloch, Carlton Covey

**Affiliations:** 1 Sports Medicine, David Grant Medical Center, Fairfield, USA

**Keywords:** prolotherapy, rehab protocol, sports ultrasound, insertional achilles tendinopathy, botulinum (botox)

## Abstract

Chronic insertional Achilles tendinopathy (IAT) is a common cause of recalcitrant heel pain. Patients present with pain localized to the Achilles tendon insertion at the calcaneus and have tenderness to palpation in this area on physical exam. Conservative management often includes an exercise prescription focusing on eccentric loading with limited dorsiflexion. Extracorporeal shockwave therapy and injection therapies including hypertonic dextrose and platelet-rich plasma (PRP) have shown some therapeutic benefit but evidence for injections is limited. IAT can often be recalcitrant to non-operative treatments, and cases are often referred for surgical debridement and decompression. Botulinum toxin A (BTX-A) has been used to treat several different musculoskeletal injuries, but there have been no published studies assessing the efficacy of BTX-A injections specifically for Achilles tendinopathy. This is a case of recalcitrant IAT treated with ultrasound-guided BTX-A.

## Introduction

Achilles tendinopathy is a chronic, overuse injury to the Achilles tendon and can be divided anatomically into insertional or mid-substance tendinopathy depending on the location of the injury. Insertional Achilles tendinopathy (IAT) is an overuse tendon injury that occurs at the distal portion of the Achilles tendon due to its insertion into the calcaneus [1]. Patients who develop IAT will often have posterior heel pain, stiffness, and swelling, with focal tenderness to palpation at the distal Achilles tendon and calcaneal insertion on physical exam [2]. While conservative treatments have shown to be effective in treating mid-substance Achilles tendinopathy, IAT is often resistant to non-operative treatment options, including strength training, eccentric exercises, orthotics, and NSAIDs, making IAT difficult to manage [2,3]. While some injection therapies including corticosteroid injections and platelet-rich plasma (PRP) injections have shown efficacy in treating mid-substance tendinopathy, these treatments have not shown the same clinical outcomes in IAT [2]. Botulinum neurotoxin is an injection therapy that has been shown to be effective in treating a variety of painful musculoskeletal conditions, including osteoarthritis, plantar fasciopathy, and lateral elbow tendinopathy. [4]. We report the first published case of IAT successfully treated with botulinum toxin A (BTX-A).

## Case presentation

**Figure 1:**
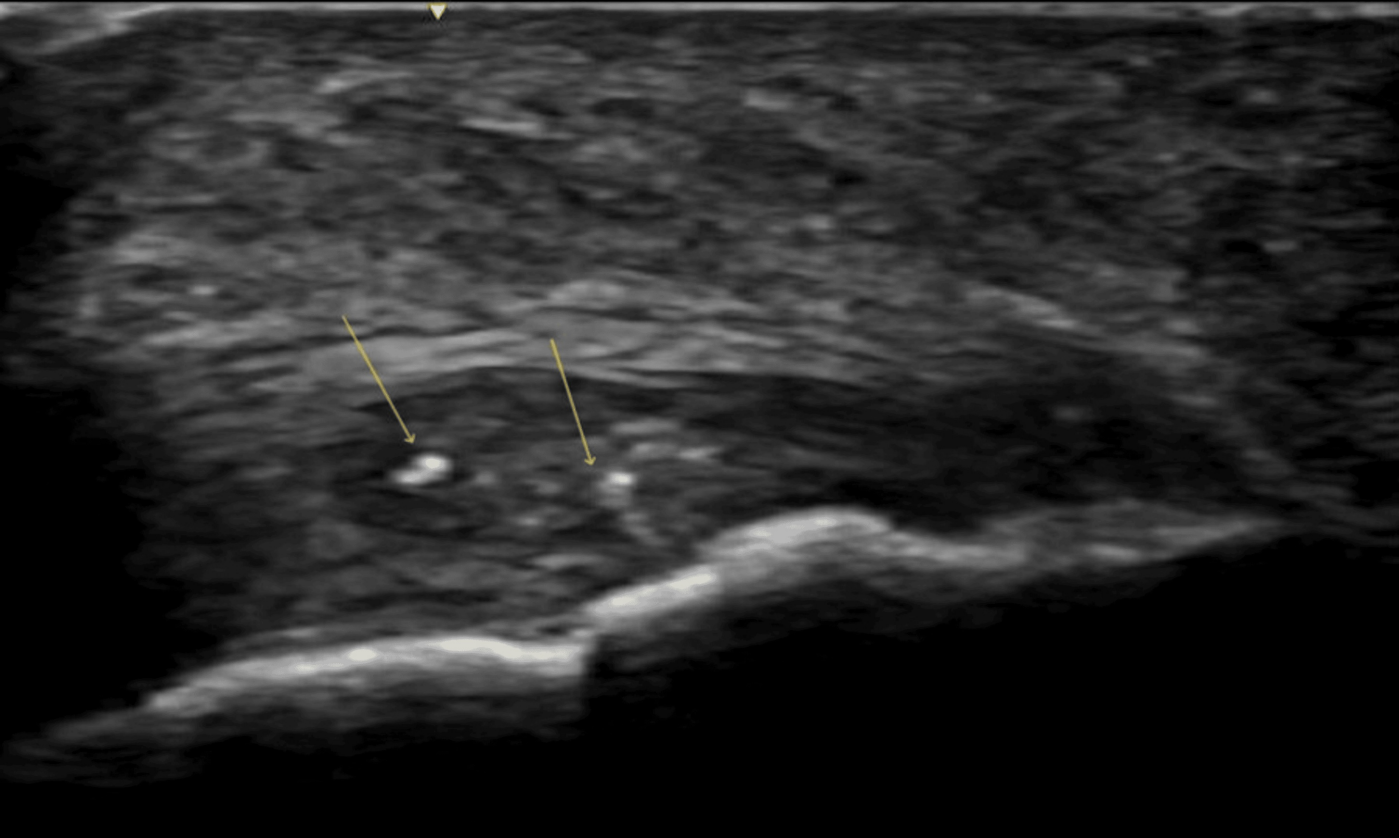
Ultrasound image of Achilles tendon in long axis Long axis ultrasound image of the Achilles tendon showing intratendinous calcifications (arrows) and alterations of echogenicity suggestive of chronic insertional tendinopathy.

A 57-year-old female presented with 12 months of progressively worsening right-sided heel pain. Pain onset was insidious, and she denied any previous mechanism of injury. The pain progressively worsened from a mild, dull ache to the point of debilitating pain with activities of daily living. She endorsed walking 150 minutes and two days of resistance training weekly, but her pain forced her to stop this exercise routine. She reported her pain as an eight out of 10 on the visual analog scale (VAS). A physical exam revealed marked tenderness to palpation over her right distal Achilles tendon insertion onto the calcaneus, exacerbated by resisted ankle plantar flexion. Thompson’s squeeze test and Achilles reflex were normal. Plain radiographs showed no bony abnormalities. Musculoskeletal ultrasound demonstrated a thickened distal Achilles tendon with multiple anechoic voids and calcifications consistent with calcific IAT (Figure 1).

The patient received a home exercise prescription focusing on eccentric Achilles tendon loading consistent with the Alfredson protocol, with specific instructions to limit dorsiflexion past neutral to avoid compressive loading [3,5]. She had no improvement after 12 weeks and was unable to progress her rehabilitation due to pain. After a discussion of additional treatment options, a series of three intratendinous dextrose prolotherapy (DPT) injections were performed using 25% dextrose spaced six weeks apart, which was consistent with previous studies [6,7]. She reported an improvement of roughly 25%, but one week after each injection, she was back to her baseline pain without sustained improvement.

**Figure 2:**
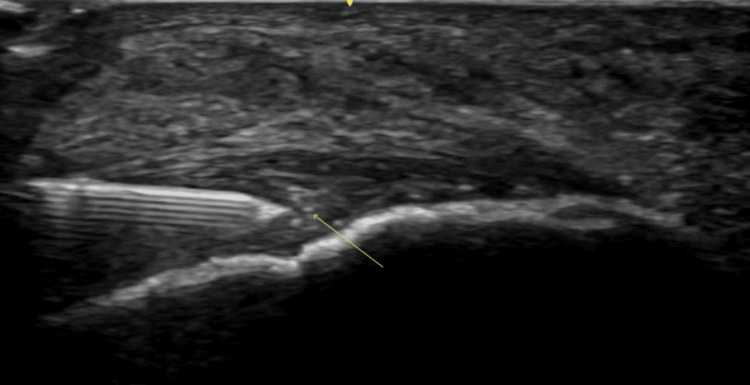
Ultrasound-guided injection of Achilles tendon Image of an ultrasound-guided injection of BTX-A using a long-axis, in-plane approach directed at the area of the most notable tendinopathic changes (arrow). BTX-A: botulinum toxin A

Subsequently, treatment options were re-addressed, with the patient deferring corticosteroid given the presence of significant tendinopathic changes. PRP injection was discussed but declined because of the patient's apprehension about the post-procedural pain. Given its successful use in the treatment of other chronic musculoskeletal conditions, the patient elected to pursue BTX-A injection [4]. After sterilizing the skin, a solution of 60 units of BTX-A mixed with normal saline for a total volume of 1 mL was injected into the right distal Achilles tendon at the calcaneal insertion under real-time ultrasound guidance (General Electric Venue Go, L4-12L-RS linear array transducer) with a 25-gauge 1.5-inch needle (Figure 2). This concentration of BTX-A was chosen based on a similar dosage used for lateral elbow tendinopathy [8]. The patient tolerated the procedure without complications and was instructed to limit activity for 48 hours and then resume activity as tolerated.

At her four-week follow-up, the patient reported the BTX-A injection provided a reduction in pain to a six on the VAS (25% improvement), and she reported that it was the first treatment that provided sustained relief. She denied paresthesia and showed no evidence of plantar flexion weakness. At eight weeks, the patient reported sustained 25% improvement and was able to ambulate and perform activities of daily living without significant discomfort. At 12 weeks, the patient was able to progress her eccentric load resistance and volume and reported continued improvement in her activity tolerance. At her six-month follow-up, she reported a reduction in pain to a four on the VAS (50% improvement), endorsed significant satisfaction with the procedure, and declined any additional injections.

## Discussion

IAT is an injury caused by overuse and cumulative microtrauma, involving the tendon-bone interface from its insertion onto the calcaneus up to 2 cm proximally [9,10]. Patients with IAT present with heel pain at the distal tendon insertion at the calcaneus, and musculoskeletal ultrasound can demonstrate a disruption in the normal fibrillar architecture, tendon hypertrophy, neovascularization on color Doppler, and bursopathy [9]. Magnetic resonance imaging (MRI) is typically reserved for recalcitrant cases to rule out other soft tissue or bony pathology, further characterize tears seen on ultrasound, or to aid in interventional or surgical planning [9].

Non-operative care is the first line for treating IAT, though traditionally less successful compared to conservative mid-substance Achilles tendinopathy programs [11]. An exercise program specifically focusing on eccentric loading with reduced dorsiflexion has shown therapeutic benefits in IAT [5,11]. Extracorporeal shock wave therapy (ESWT) is another treatment option that has shown therapeutic benefit in IAT, with several clinical trials demonstrating similar results with a ≥2-point decrease in pain [10,11]. There is a consensus to avoid intratendinous corticosteroid injections due to concerns for exacerbating tendon degeneration and increasing the risk of rupture [9,10]. Hypertonic dextrose and PRP injections have shown therapeutic benefits, but the evidence is limited [6,7,11].

BTX-A is a potent neuromuscular blocking agent that has been shown to decrease pain in multiple musculoskeletal conditions [4]. Though the use of BTX-A for the treatment of chronic musculoskeletal conditions has been well-studied, its use for these conditions is considered off-label by the Food and Drug Administration (FDA). The use of BTX-A for painful musculoskeletal conditions is supported by two hypothesized mechanisms: a protective, paralyzing effect on muscles that reduces tendon tension and an analgesic effect due to inhibition of the neurotransmitters involved in pain transmission [12]. Adverse effects may include transient weakness of the injected muscle [4,8,12].

To the authors' knowledge, this is the first case to demonstrate the safe and successful use of ultrasound-guided BTX-A injection to treat recalcitrant IAT. In this case, the patient had confirmed IAT evidenced by exam and sonographic findings. The patient did not improve with an eccentric rehabilitation program and had a suboptimal response to DPT injections. As the patient declined other injection modalities and surgical referral, through shared decision-making, BTX-A injection was performed. The BTX-A injection was the only treatment that provided a sustained, significant improvement in her baseline pain. The BTX-A showed no adverse effects, and the patient was able to resume her daily activities without significant pain and advance her rehabilitation program. In IAT cases refractory to conservative management, BTX-A may be a safe and effective non-operative treatment option, though further studies are warranted.

## Conclusions

IAT can be difficult to manage, as it is often refractory to various treatment modalities. This case demonstrates that BTX-A injection therapy may be a safe and effective treatment option for patients with recalcitrant IAT, though additional studies are needed to assess the optimal dosing and potential risks that may be associated with BTX-A injections performed within the Achilles tendon.
